# Metabolism and Pharmacokinetic Study of the Boron-Containing Prodrug of Belinostat (ZL277), a Pan HDAC Inhibitor with Enhanced Bioavailability

**DOI:** 10.3390/ph12040180

**Published:** 2019-12-08

**Authors:** Changde Zhang, Shanchun Guo, Qiu Zhong, Qiang Zhang, Ahamed Hossain, Shilong Zheng, Guangdi Wang

**Affiliations:** RCMI Cancer Research Center and Department of Chemistry, Xavier University of Louisiana, New Orleans, LA 70125, USA; czhang1@xula.edu (C.Z.); sguo@xula.edu (S.G.); qzhong@xula.edu (Q.Z.); qzhang1@xula.edu (Q.Z.); hahamed@xula.edu (A.H.)

**Keywords:** tumor, ZL277 metabolism, belinostat, HDAC inhibitor, pharmacokinetics

## Abstract

ZL277 is a prodrug of belinostat with enhanced bioavailability and efficacy as a pan histone deacetylase (HDAC) inhibitor. In this study, we investigated the metabolism and pharmacokinetics of ZL277 in liver S9 fractions, liver microsomes, liver cytosol, and in mice. Metabolic products were identified and quantified by a combination of liquid chromatography and tandem mass spectrometry. The in vitro metabolic profile of ZL277 includes ZL277-B(OH)_2_-452, the major oxidative metabolite ZL277-OH-424, the active ingredient belinostat, belinostat amide, belinostat acid, and methylated belinostat in liver S9 fractions. Both ZL277-OH-424 and belinostat underwent further glucuronidation in liver microsome, whereas only ZL277-OH-424, but not belinostat, underwent some level of sulfation in rat liver cytosols. These metabolites were examined in plasma and in a breast tumor model in vivo. They were also examined in urine and feces from mice treated with ZL277. The pharmacokinetic study of ZL277 showed the parameters of active drug belinostat with a half-life (t_1/2_) of 10.7 h, an area under curve value (AUC) of 1506.9 ng/mL*h, and a maximum plasma concentration (C_max_) of 172 ng/mL, reached 3 h after a single dose of 10 mg/kg. The hydrolysis product of the prodrug, ZL277-B(OH)_2_-452 showed an AUC of 8306 ng/mL*h and C_max_ of 931 ng/mL 3 h after drug administration.

## 1. Introduction

**Figure 1:**
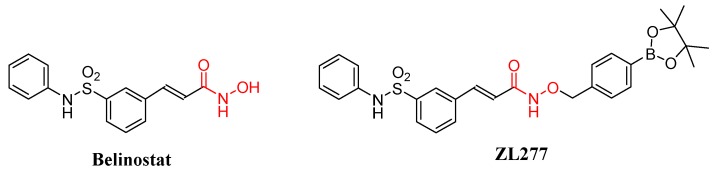
Molecular structures of belinostat and ZL277.

A number of histone deacetylase (HDAC) inhibitors have been tested in clinical trials for the treatment of various types of tumors [1,2], neurodegenerative disorders [3], inflammation disorders [4], and cardiovascular disease [5]. Belinostat is the first of four FDA-approved HDAC inhibitors for the treatment of relapsed/refractory peripheral T-cell lymphoma [6]. Moreover, HDAC inhibitors (including belinostat) have been found to augment the response to PD-1 immunotherapy in lung adenocarcinoma [7] and melanoma [8], as stand-alone agents or in combination with immunotherapeutic approaches [9]. However, belinostat and other HDAC inhibitors have very limited therapeutic outcome for the treatment of nonhematological cancers in completed clinical trials [2]. To expand the potential clinical utilities of HDAC inhibitors, we have designed and synthesized the boron-containing prodrug of belinostat (ZL277), which has been shown to inhibit tumor growth in a breast cancer xenograft model with enhanced bioavailability and efficacy (Figure 1) [10]. 

ZL277 is a chemically modified belinostat in which the labile hydroxyl group of the hydroxamic acid is conjugated and sealed with a p-boronate benzyl moiety. The p-boronate benzyl moiety was designed to be more efficiently oxidized inside cancer cells containing higher intracellular concentrations of H_2_O_2_ compared to normal cells [11,12], thereby facilitating a self-immolation release of the belinostat molecule. In vitro, ZL277 showed slightly weaker antiproliferative activities than belinostat in two cancer cell lines, MDA-MB-231 and MCF-7, as expected from the incomplete conversion from the prodrug form to the active ingredient (belinostat). However, in vivo, ZL277 exhibited greater efficacy than belinostat in blocking the growth of MCF-7 tumor xenograft in mice. ZL277 caused tumor regression, while belinostat only inhibited tumor growth [10].

The pharmacokinetics (PK) and metabolism of belinostat have been extensively studied [13,14,15,16,17,18,19]. It has been reported that belinostat undergoes rapid glucuronidation, catalyzed by UGT1A1, -1A3, -1A8, -2B4, and -2B7 [13,14,18]. The main metabolic pathway of belinostat is through glucuronidation, mediated primarily by UGT1A1, and the predominant site of belinostat glucuronidation was found at the hydroxyl position, while other minor metabolites are belinostat amide, belinostat acid, methyl belinostat, belinostat glucoside and 3-(anilinosulfonyl)-benzenecarboxylic acid. These metabolites of belinostat are inactive or very weakly active in clonogenic assays. These observations help to explain the poor bioavailability and limited therapeutic outcome of belinostat in vivo. 

In this study, we investigated the in vitro and in vivo metabolism and pharmacokinetics of ZL277 by incubating liver S9 fractions, microsomes, and liver cytosols and using rodents treated with ZL277 via intraperitoneal (IP) injection. Liquid chromatography, coupled with high-resolution tandem mass spectrometry, was employed to analyze ZL277 and its related metabolic products in incubation mixtures, mice plasma, tumor tissue, urine, and feces samples. 

## 2. Results and Discussion

### 2.1. In Vitro Metabolism of ZL277 in Liver S9 Fraction

**Figure 2:**
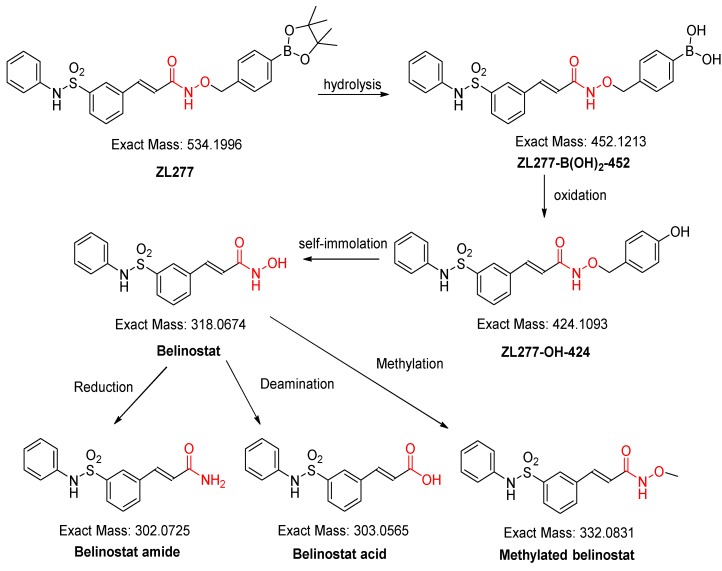
The oxidative metabolic pathways of ZL277 in liver S9 fraction.

**Figure 3:**
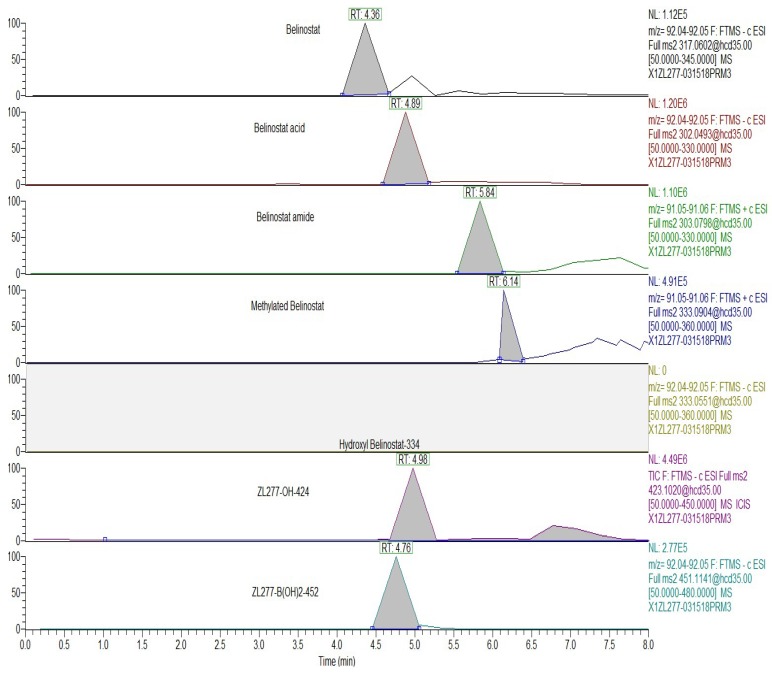
Selected ion chromatograms of ZL277 metabolites from incubation with rat liver S9 fraction.

The liver S9 fractions contain both the microsome component and the cytosol component, with enzymes responsible for oxidation and reduction reactions. NADPH is necessary for maintaining the electron balance in xenobiotic oxidation reactions. ZL277 was subjected to various redox and methylation reactions under aerobic conditions by incubating ZL277 with the rat liver S9 fraction for 1 h in the presence of NADPH. Using a high-resolution mass spectrometer, six metabolites of ZL277 were detected and identified from the incubation mixture (Figure 2). The hydrolysis of ZL277 formed ZL277-B(OH)_2_-452, which was oxidized and de-boronated into ZL277-OH-424, which was then further metabolized to belinostat. Belinostat was quickly reduced to belinostat amide, de-aminated into belinostat acid, and methylated into methylated belinostat (Figure 2 and Figure 3). The metabolites downstream of belinostat are consistent with the reported metabolic profile of belinostat in a phase 1 clinical trial [14].

### 2.2. In Vitro Glucuronidation of ZL277

**Figure 4:**
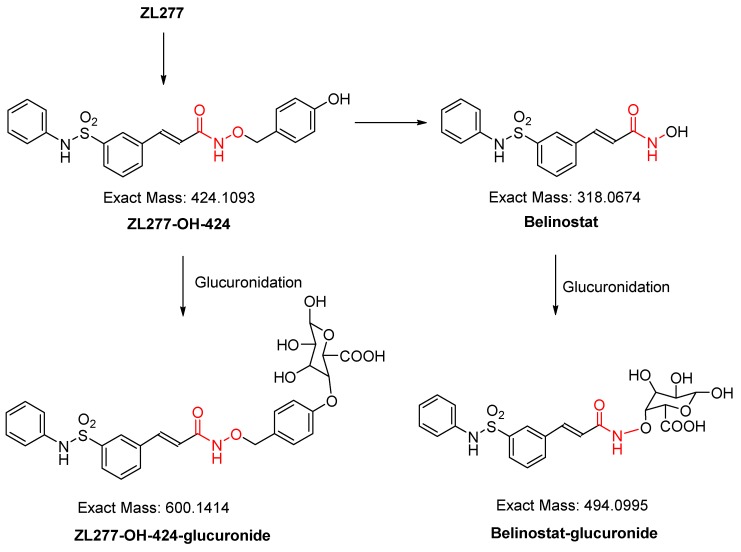
The glucuronidation of ZL277 from incubation with rat liver microsomes and uridine diphosphate glucuronic acid.

**Figure 5:**
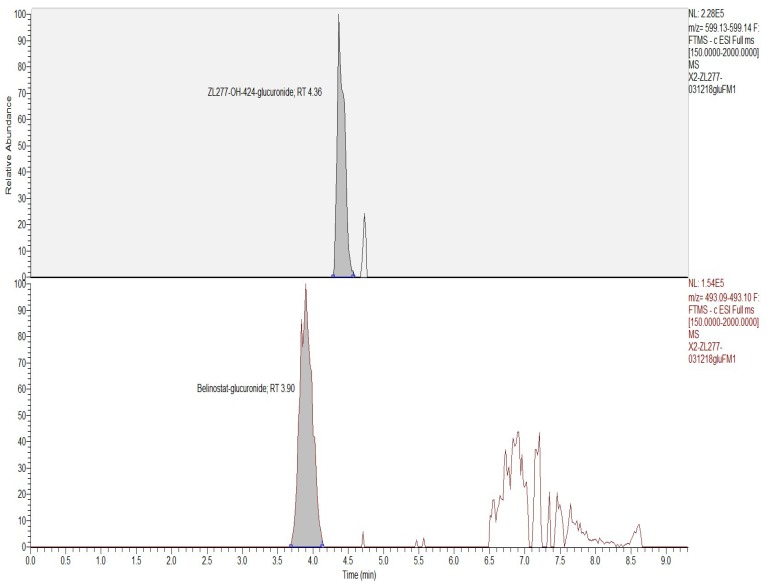
Selected ion chromatograms of glucuronidation metabolites of ZL277 from incubation with liver microsomes and uridine diphosphate glucuronic acid for 1 h.

Glucuronidation is a major phase II biotransformation reaction in belinostat metabolism [13]. After incubation with rat liver microsomes and uridine diphosphate-glucuronic acid (UDPGA) for 1 h, the glucuronidation products of ZL277 in vitro were examined. Both glucuronidation products of belinostat and ZL277-OH-424 were detected (Figure 4 and Figure 5). Belinostat–glucuronide was observed at 3.90 min in the chromatograph and ZL277-OH-424-glucuronide was detected at a retention time of 4.36 min.

### 2.3. In Vitro Sulfate Conjugate Formation of ZL277

**Figure 6:**
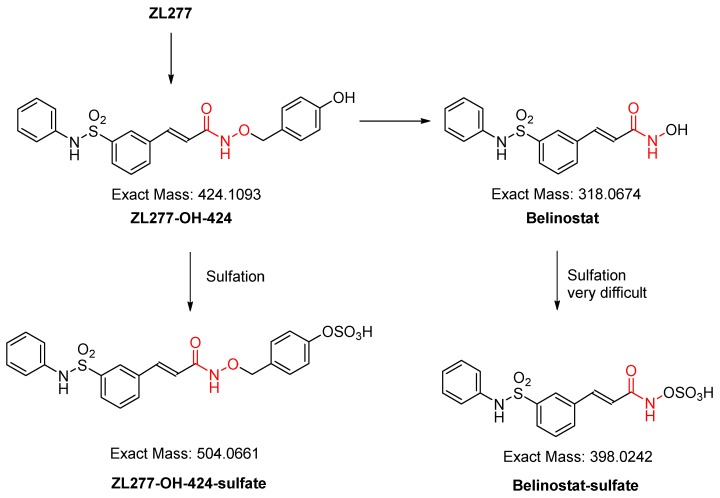
The sulfation metabolism of ZL277 from incubation with rat liver cytosols and 3’-phosphoadenosine-5’-phosphosulfate.

**Figure 7:**
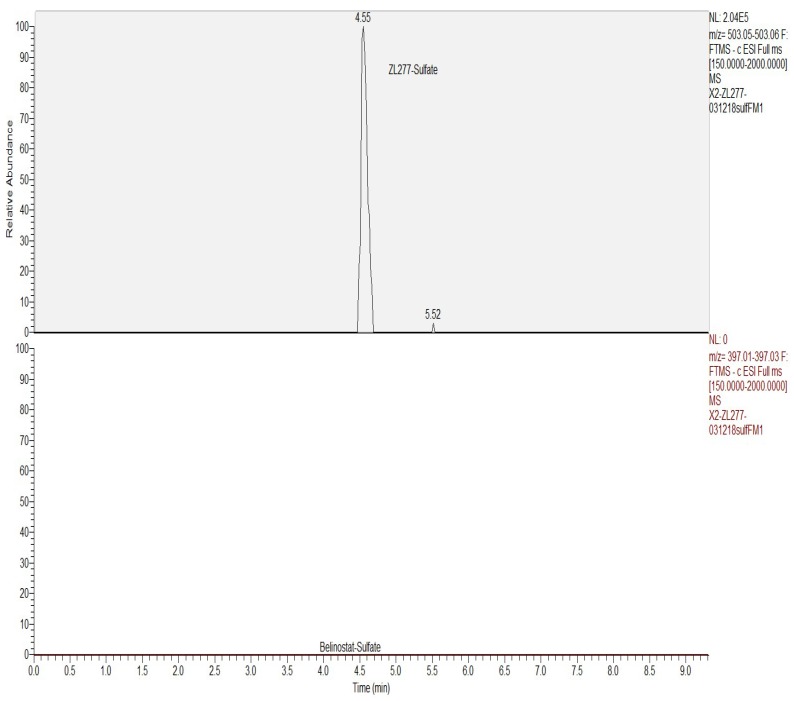
Selected ion chromatograms of the incubation mixture of ZL277 in liver cytosol and 3’-phosphoadenosine-5’-phosphosulfate.

Sulfation is another important phase II biotransformation reaction for many drugs. After incubation with rat liver cytosol and 3’-phosphoadenosine-5’-phosphosulfate (PAPS) for 30 min, the sulfate of ZL277-OH-424 was detected (Figure 6 and Figure 7). ZL277-OH-424-sulfate appeared at 4.55 min in the LC chromatograph. No peak of belinostat–sulfate was found in the incubation mixture. Although several aromatic hydroxamic acids with an aromatic ring substituted on the nitrogen were reported to precede sulfation in the liver cytosol [20,21], their sulfation rate could not compete with the sulfation of ZL277-OH-424. 

### 2.4. Pharmacokinetics and in Vivo Metabolites of ZL277 in Mice Plasma

**Figure 8:**
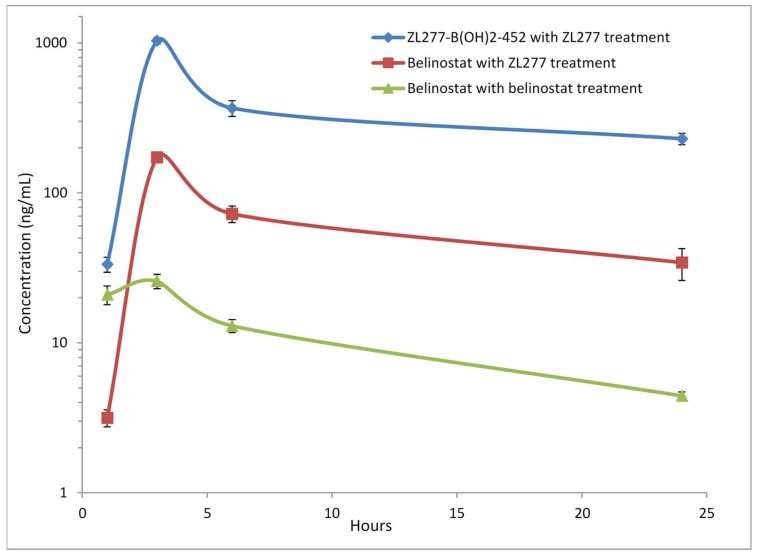
Mean plasma concentration time profile after a single dose of 10 mg/kg IP injection with ZL277 or belinostat.

The bioavailability of a drug reflects the fraction of an administered dose of this drug that reaches systemic circulation in blood. To determine the bioavailability of ZL277 and its active metabolite belinostat, we conducted pharmacokinetic studies of ZL277 in mice. After a single dose of 10 mg/kg ZL277 or belinostat by IP injection, blood samples were collected from mice and analyzed for concentration of belinostat and the prodrug at 1, 3, 6, and 24 h time points after drug administration (Figure 8). As shown in Table 1, ZL277 afforded 172.67 ng/mL peak concentration of belinostat 3 h after administration, 6.7-fold higher than the 25.78 ng/mL achieved by belinostat at a single dose of 10 mg/kg. At 24 h, the ZL277 treatment group had 34.31 ng/mL concentration of belinostat. Moreover, the predominant form in mice plasma is ZL277-B(OH)_2_-452, the corresponding free boronic acid of ZL277, with belinostat accounting for 10%~20% of the total drug and metabolite concentration. The maximum concentration of ZL277-B(OH)_2_-452 reached 930.77 ng/mL, about forty times that of the maximum concentration (25.78 ng/mL) achieved by belinostat. With treatment of ZL277, mice showed a slightly longer half-life of belinostat, at 10.7 h, with the AUC of belinostat at 1.51 µg/mL h, which is 5-fold higher than in mice treated with belinostat IP injection. As well as this, the AUC of ZL277-B(OH)_2_-452 was at 8.31 µg/mL h, or 28.6-fold greater than the AUC of belinostat in mice treated with belinostat IP injection (Table 2). ZL277-OH-424 was detected in plasma at slightly above our instrument detection limit and was not quantified in this PK study. These observations provide definitive evidence that the bioavailability of ZL277 is superior compared to belinostat. The above data were obtained from the same dose of 10 mg/kg for both ZL277 and belinostat. Considering that the dose of 10 mg/kg ZL277 corresponds to 18.7 µmol/kg, and the dose of 10 mg/kg belinostat corresponds to 31.4 µmol/kg, due to their different molecular weights, the bioavailability advantage of ZL277 over belinostat was even more pronounced when the molar normalization of the dosage was taken into account, where belinostat doses of 18.7 or 5.96 mg/kg were used.

The metabolites discovered in the plasma samples from mice treated with ZL277 are listed in Table 3. ZL277-B(OH)_2_-452, ZL277-OH-424, belinostat, belinostat amide, belinostat acid, methylated belinostat, and belinostat-glucuronide were detected in mouse plasma. It is interesting that the quantity of ZL277-OH-424 inside the plasma samples was only slightly above the detectable level, suggesting a short life of the intermediate. Neither ZL277-OH-424-sulfate nor ZL277-OH-424-glucuronate were detected in the plasma in vivo; conversely, they were found in vitro incubation of ZL277 with microsomes or cytosols. Belinostat–glucuronide is one significant metabolite in plasma from ZL277-treated mice, consistent with that of belinostat intravenous injection (IV)-treated patients [22].

### 2.5. Metabolites of ZL277 in Tumor Tissues

In the breast tumor xenograft in mice treated with ZL277, we found ZL277-B(OH)_2_-452, ZL277-OH-424, and belinostat and its derivatives (belinostat amide, belinostat acid, and methylated belinostat). In contrast, no glucuronide or sulfate of belinostat or ZL277 were detected in the breast tumor samples (Table 3). We measured the concentration of belinostat and its prodrugs ZL277-B(OH)_2_-452 and ZL277-OH-424 in breast tumor tissues (Table 4), sampled 4 h after the final treatment. The level of belinostat in tumor tissues (223.1 ng/g) treated with ZL277 was slightly higher than those treated with belinostat (172.1 ng/g) at the same dose. Tumor tissues from the ZL277 treatment group also contained 2706.1 ng/g ZL277-B(OH)_2_-452, and 166.2 ng/g ZL277-OH-424, which are precursors of belinostat. If we consider their molecular weight difference—the molecular weight (MW) of ZL277 (534.1 g/mol) is significantly higher than that of belinostat (318.3 g/mol)—the dose of belinostat (10 mg/kg or 31.4 µmol/kg) treatment was actually 1.68 times that of ZL277 (10 mg/kg or 18.7 µmol/kg), based on their molarity. The difference in belinostat concentration in breast tumor tissue should be larger than the data shown in this table if the animals were treated with equal molar quantities. 

### 2.6. The Metabolites of ZL277 in Urine Samples from Mice Treated with ZL277

**Figure 9:**
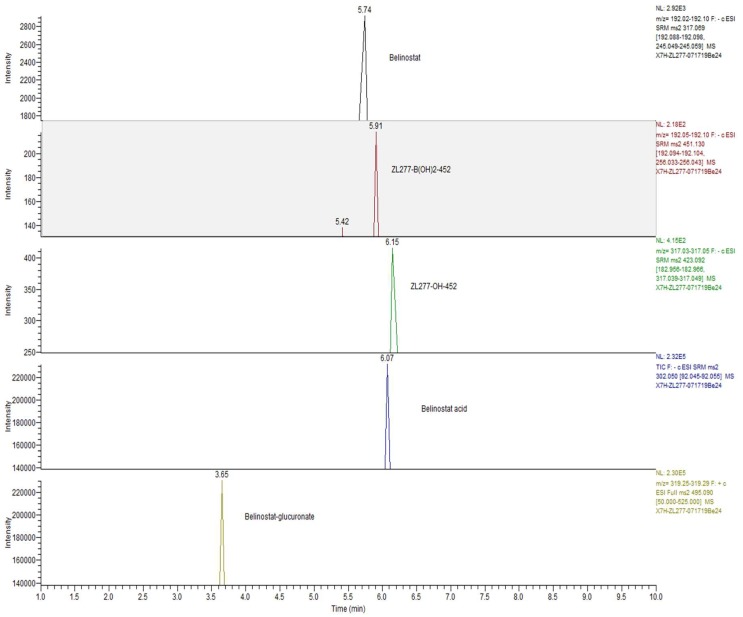
UPLC-MS chromatogram of the main ZL277 metabolites in urine samples collected at 8 h post-dosage.

In the urine of mice treated with ZL277, ZL277-B(OH)_2_-452, ZL277-OH-424, belinostat and its derivatives were detected (Table 3 and Figure 9). These derivatives include belinostat amide, belinostat acid, methylated belinostat, and belinostat–glucuronide. Among them, belinostat, ZL277-B(OH)_2_-452, ZL277-OH-424, and belinostat acid are major components, where belinostat–glucuronide and belinostat acid are dominant components with much greater intensities. No sulfate of belinostat or sulfate of ZL277 were detected. The majority of ZL277 was excreted as belinostat–glucuronide and belinostat acid in urine. Most of these metabolites were cleared out within 24 h of IP administration and were below detection limit from the urine samples collected 24 h after IP administration. 

### 2.7. The Metabolites of ZL277 in Fecal Samples from Mice Treated with ZL277

**Figure 10:**
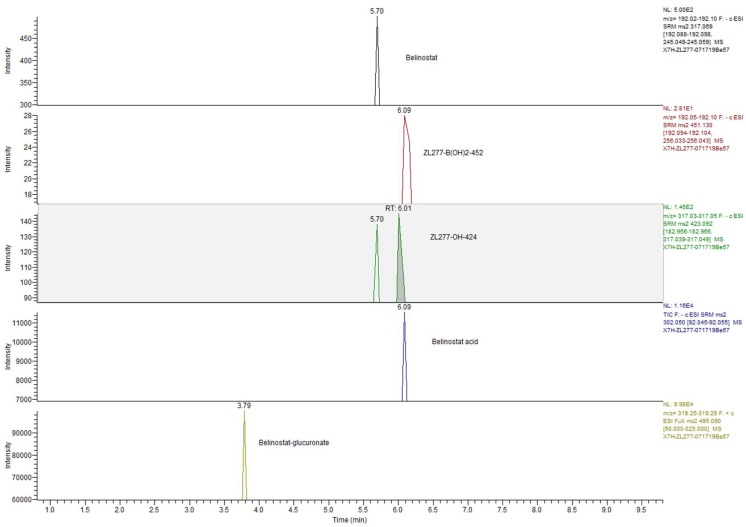
UPLC-MS chromatogram of main ZL277 metabolites in feces collected 8h after one dose of 10 mg/kg IP injection of ZL277.

As in mouse urine samples, the seven metabolites were all found in the feces of mice treated with ZL277. ZL277-B(OH)_2_-452, ZL277-OH-424, belinostat, belinostat acid, and belinostat-glucoronate are major metabolites in fecal samples. ZL277 was excreted dominantly in the forms of belinostat–glucuronide and belinostat acid in the feces (Table 3 and Figure 10). Most of these metabolites were present in fecal samples collected within 24 h after IP administration, except a small quantity of belinostat–glucuronide, which was detected in the feces collected 24 h after dosage. 

## 3. Experimental Section

### 3.1. Chemicals

Rat liver S9 fractions, microsomes and cytosols were purchased from Sekisui XenoTech. NADPH solution A, NADPH solution B, UGT Reaction Mix solution A, and UGT Reaction Mix solution B were purchased from Corning Gentest. 3’-Phosphoadenosine-5’-phosphosulfate (PAPS) was purchased from R&D Systems. ZL277, ZL277-B(OH)_2_-452, and ZL277-424-OH were synthesized in our lab. All other chemicals and reagents were obtained from Fisher Scientific.

### 3.2. Liver S9 Fraction Metabolism of ZL277

The incubation of ZL277 with liver S9 fractions followed the procedures of incubation with liver microsomes, as described previously [23]. The pre-incubation solution consisted of 30 µL of potassium phosphate buffer (pH 7.4; 10X), 241.5 µL water, 15 µL of NADPH solution A, 3 µL of NADPH solution B, 7.5 µL rat liver S9 fractions. After incubation at 37 °C for 5 min in a 1.5 mL microcentrifuge vial, 3 µL of 10 mM ZL277 was added, mixed, and incubated at 37 °C for another 60 min in the incubator. Then, 300 µL MeOH was added to terminate the reaction. After centrifugation, the supernatant was analyzed on the UHPLC, coupled to a Q-Exactive high-resolution mass spectrometer. 

### 3.3. Glucuronidation of ZL277 in Liver Microsomes

The incubation of ZL277 with microsomes followed the similar procedures for studying glucuronidation of ZB716 [23]. The mixture of 205.5 µL water, 24 µL of UGT Reaction Mix solution A, 60 µL of UGT Reaction Mix solution B, and 7.5 µL rat liver microsomes was incubated at 37 °C for 5 min. 3 µL of 10 mM ZL277 was added, mixed, and incubated at 37 °C for another 60 min. 300 µL MeOH was added to terminate the reaction. After centrifugation, the supernatant was analyzed on the Q-Exactive HRMS. 

### 3.4. Sulfation of ZL277 in Liver Cytosols

The incubation of ZL277 with liver cytosols followed a similar procedure to the study of sulfation of ZB716 [23]. After termination of incubation with 300 µL MeOH, the final mixture was centrifuged. The supernatant was analyzed on the UHPLC-Q-Exactive instrument. 

### 3.5. Sample Collection of Plasma, Urine, and Feces in Metabolite Study and Pharmacokinetics Study

Four to six-week-old female ovariectomized Nu/Nu mice (Genotype: Homozygous genotype, homozygous for Foxn1<nu>), purchased from Charles River Laboratories (Wilmington, MA, USA), were used for the study of ZL277 metabolites in vivo. Six mice in each group were given ethanol-dissolved ZL277 and belinostat, at a single dose of 10 mg/kg by intraperitoneal injection (IP). After IP administration, venous blood samples were collected at 1, 3, 6, and 24 h time points. Urine and fecal samples were collected for the time duration of 0–8 h, 8–24 h, 24–32 h, and 32–48 h after IP administration.

### 3.6. Sample Collection of Breast Tumors in Nude Mice Model

Four to six week old female ovariectomized Nu/Nu mice, implanted subcutaneously with 17β-Estradiol pellets, were injected with MCF-7 cells harvested in the exponential growth phase to grow the breast tumors. On day 15 after tumor formation, the mice were treated with vehicle, belinostat, at 10 mg/kg/day, or ZL277 at 10 mg/kg/day, by subcutaneous injection. On the last day of the study, the tumors were collected and frozen for analysis.

### 3.7. Analysis of Metabolites on HR Mass Spectrometer

The samples from in vitro incubations were run on a Hypersil GOLD C18 column (1.8 µm, 2.1 × 50 mm) with UHPLC ultimate 3000 from Dionex coupled with one Q-Exactive HR mass spectrometer (Thermo Scientific, Waltham, MA, USA). The gradient started at 20% mobile phase B (Acetonitrile with 0.05% formic acid) and 80% mobile phase A (water with 0.05% formic acid), increased to 100% B in 4.5 min at 0.3 mL/min, stayed for 5 min, then came back to 30% B until equilibration. PRM mode or full scan mode was used to identify the interested metabolites. 

### 3.8. Analysis of Metabolites on TSQ Mass Spectrometer

Plasma, urine, or feces samples were processed following protocols published previously [23]. These samples were injected on the Hypersil GOLD C18 column (1.8 µm, 2.1 × 50 mm) with UHPLC ultimate 3000 from Dionex, coupled with a TSQ Voltage mass spectrometer, to quantitate the concentration of the main metabolites of ZL277. The 10 µL samples were run with the gradient starting at 0.3 mL/min from 2% mobile phase B (acetonitrile with 0.05% formic acid) and 98% mobile phase A (water with 0.05% formic acid) until 1 min, up to 30% B at 3 min, 45% B at 4.8 min, 100% B at 5.5 min, then back to 30% B until equilibration. SRM mode was used to detect ZL277 (pos 535.21→134.97), ZL277-B(OH)_2_-452 (neg 451.13→256.04), ZL277-424-OH (neg 423.07→317.04), belinostat (neg 317.02→245.05) or (pos 319.08→93.08), belinostat amide (pos 303.08→91.05), belinostat acid (neg 302.05→92.05), methylated belinostat (pos 333.09→239.92). The product scan in MS/MS mode was used to detect belinostat–sulfate (neg 397.02→97.02), and belinostat–glucoronate (pos 495.09→319.27). The TSQ Voltage was set at spray voltage at 3200 V, vaporizer temperature at 365 °C, sheath gas pressure at 33 psi, auxiliary gas pressure at 10 psi, and capillary temperature at 350 °C. Additional experimental details can be found in Figures S1 and S2, and Tables S1–S8.

All procedures involving the animals were conducted in compliance with State and Federal laws, standards of the U.S. Department of Health and Human Services, and guidelines established by Xavier University Animal Care and Use Committee. The facilities and laboratory animals program of Xavier University of Louisiana are accredited by the Association for the Assessment and Accreditation of Laboratory Animal Care.

## 4. Conclusions

In summary, the metabolic profile of ZL277 in vitro consisted of ZL277-B(OH)_2_-452, ZL277-424-OH, belinostat, belinostat amide, belinostat acid, methylated belinostat, ZL277-424-OH–glucuronide, belinostat glucuronide, and ZL277-424-OH-sulfate. ZL277 was quickly hydrolyzed to form the boronic acid form of the prodrug, and was not found in vitro. The pharmacokinetics of ZL277 showed far superior bioavailability to belinostat. The xenograft breast tumor from mice treated with ZL277 showed the accumulation of belinostat and its precursors ZL277-B(OH)_2_-452 and ZL277-OH-424, with concentrations over 15-fold of that of belinostat found in tumor tissues of mice treated with belinostat. ZL277 was mainly excreted as ZL277-B(OH)_2_-452, ZL277-424-OH, belinostat, belinostat acid, and belinostat glucoronate. Belinostat acid and belinostat glucuronide were the two dominant components in urine and feces. Our preliminary study revealed that ZL277 is more efficacious than belinostat in vivo [10], not only inhibiting the growth of tumors but also significantly reducing tumor volumes in an MCF-7 xenograft tumor model, owing to its superior biocompatibility and drug concentration in the tumor tissue. We will test more solid tumors in future studies.

## Figures and Tables

**Table 1:** Pharmacokinetic parameters of ZL277 and belinostat in mice after an intraperitoneal injection (IP) of 10 mg/kg ZL277 (18.7 µmol/kg) or belinostat (31.4 µmol/kg).

Time Point (h)	ZL277 (Molecular Weight (MW) = 534.1)		Belinostat (MW = 318.1)
	Belinostat (ng/mL)	ZL277-B(OH)_2_-452 (ng/mL)	Belinostat (ng/mL)
1	3.16 ± 0.22	36.75 ± 3.85	20.94 ± 2.99
3	172.67 ± 5.63	930.77 ± 50.07	25.78 ± 2.80
6	72.54 ± 5.11	367.62 ± 44.71	14.28 ± 1.29
24	34.31 ± 4.54	229.35 ± 20.23	5.32 ± 0.28

**Table 2:** Pharmacokinetics parameters of belinostat and ZL277-B(OH)_2_-452 in mice after an intraperitoneal injection (IP) of 10 mg/kg ZL277 (18.7 µmol/kg) or belinostat (31.4 µmol/kg).

IP Drug	Belinostat (MW = 318.1)	ZL277 (MW = 534.1)	
	Belinostat	Belinostat	ZL277-B(OH)_2_-452
t_1/2_ (h)	10.18	10.72	13.18
C_max_ (ng/mL)	25.8	172.7	930.8
AUC (µg/mL∙h)	0.29	1.51	8.31

**Table 3:** In vivo metabolites of ZL277 in mice after intraperitoneal injection (IP).

	Plasma	Tumor	Feces	Urine	RT (min)
ZL277	−	−	−	−	
ZL277-B(OH)_2_-452	+	+	+	+	5.90
ZL277-OH-424	+	+	+	+	6.05
Belinostat	+	+	+	+	5.70
Belinostat amide	+	+	+	+	5.17
Belinostat acid	+	+	+	+	6.09
Methylated belinostat	+	+	+	+	6.55
ZL277-OH-424-sufate	−	−	−	−	
Belinostat–sulfate	−	−	−	−	
ZL277-OH-424-glucuronide	−	−	−	−	
Belinostat–glucuronide	+	−	+	+	3.75

**Table 4:** Drug and metabolite concentrations in xenograft tumor tissues from mice treated with ZL277 (18.7 µmol/kg) or belinostat (31.4 µmol/kg) at 10 mg/kg IP injection treatment 4 h after last treatment.

ZL277			Belinostat
Belinostat (ng/g)	ZL277-OH-424 (ng/g)	ZL277-B(OH)_2_-452 (ng/g)	Belinostat (ng/g)
223.1± 29.2	166.2 ± 45.3	2706.1 ± 152.5	172.1 ± 28.9

## Supplementary Materials

The following are available online at https://www.mdpi.com/1424-8247/12/4/180/s1, Figure S1: UPLC MS/MS extracted Chromatograms (A) SRM transition of free belinostat, ZL277-B(OH)2-452, and ZL277-OH-424 standard; (B) a blank mice plasma sample (no belinostat/ZL277); (C) SRM transitions of belinostat, ZL277-B(OH)2-452, and ZL277-OH-424 in mice plasma 3 h after 10 mg/kg ZL277 IP injection treatment; (A) a blank mice breast tumor control sample (without ZL277 or belinostat treatment); (B) SRM transition of belinostat, ZL277-B (OH)2-452, and ZL277-OH-424 in breast tumors at one month after 10 mg/kg ZL277 daily IP injection treatment; Table S1. PRM workflow parameters for the Q-Exactive mass spectrometer; Table S2. Full MS workflow parameters for the Q-Exactive mass spectrometer; Table S3. NSI tune parameters for the Q-Exactive mass spectrometer; Table S4. Instrument tune parameters for the TSQ Vantage mass spectrometer; Table S5. Detected limitations and correlation equations of belinostat, ZL277-B(OH)2-452, and ZL277-OH-424 for plasma samples during validation; Table S6. Detected limitations and correlation equations of belinostat, ZL277-B(OH)2-452, and ZL277-OH-424 for breast tissue during validation; Table S7. Between-run precision and accuracy of Quality control samples for plasma sample measurement during validation; Table S8. Between-run precision and accuracy of Quality control samples for breast sample measurement during validation.
