# Supplementary material for: Metabolism and Pharmacokinetic Study of the Boron-Containing Prodrug of Belinostat (ZL277), a Pan HDAC Inhibitor with Enhanced Bioavailability
Source: Pharmaceuticals (Basel). 2019 Dec 8;12(4):180. doi: 10.3390/ph12040180 (PMC6958523; doi:10.3390/ph12040180)
Supplement: Supplementary file 1 [file pharmaceuticals-12-00180-s001.pdf]

Supplemental Material

Metabolism and pharmacokinetic study of the boron-containing prodrug of belinostat (ZL277), a pan HDAC inhibitor with enhanced bioavailability

Changde Zhang[§], Shanchun Guo[§], Qiu Zhong, Qiang Zhang, Ahamed Hossain, Shilong Zheng*, and Guangdi Wang*

RCMI Cancer Research Center and Department of Chemistry, Xavier University of Louisiana, New Orleans, LA 70125

Contents

**Table S1**	**Page 2**
**Table S2**	**Page 2**
**Table S3**	**Page 3**
**Table S4**	**Page 3**
**Table S5**	**Page 3**
**Table S6**	**Page 4**
**Table S7**	**Page 4**
**Table S8**	**Page 4**
**Figure S1**	**Page 5**
**Figure S2**	**Page 7**
**Sample Preparation Protocols**	**Page 8**

Table S1. PRM workflow parameters for the Q-Exactive mass spectrometer.

Workflow			
**Negative PRM**		**Positive PRM**	
**General**		**General**	
Run time	0-9.3min	Run time	0-9.3min
Polarity	negative	Polarity	positive
In-source-CID	0.0ev	In-source-CID	0.0ev
Default charge	1	Default charge	1
Inclusion	on	Inclusion	on
**MS^2**		**MS^2**	
Microscans	1	Microscans	1
Resolution	17500	Resolution	17500
AGC Target	2e4	AGC Target	2e4
Max Inject Time (ms)	100ms	Max Inject Time (ms)	100ms
Loop count	1	Loop count	1
Isolation window	4.0 m/z	Isolation window	4.0 m/z
Isolation offset	0.0 m/z	Isolation offset	0.0 m/z
(N)CE/stepped nce	35	(N)CE/stepped nce	35
Spectrum data t	Profile	Spectrum data t	Profile

Table S2. Full MS workflow parameters for the Q-Exactive mass spectrometer.

Workflow	
**Properties of Full MS-SIM**	
**General**	
Run time	0-9.3min
Polarity	negative
In-source-CID	0.0ev
**Full MS-SIM**	
Microscans	1
Resolution	17500
AGC Target	2e6
Max Inject Time (ms)	200ms
Number of scan	1
Scan range	100-1000 m/z
Spectrum data t	Centroid

Table S3. NSI tune parameters for the Q-Exactive mass spectrometer.

**Parameters of NSI source**	
**NSI source**	
Sheath gas flow rate	10
Auxgas flow rate	0
Sweep gas flow rate	0
Spray voltage (kv)	1.50
Spray current (μA)	
Capillary temperature (ºC)	250
S-lens RF level	50.0

Table S4. Instrument tune parameters for the TSQ Vantage mass spectrometer.

**Parameters of instrument tune setup**	
Spray voltage	3200
Vaporizer temperature (ºC)	365
Sheath gas pressure (PSI)	33
Ion sweep gas pressure (PSI)	1.5
Aux gas pressure (PSI)	10
Capillary temperature (ºC)	350
S-lens RF amplitude	51

Table S5. Detect limitations and correlation equations of belinostat, ZL277-$B(OH)_2$-452, and ZL277-OH-424 for plasma samples during validation.

	Belinostat	ZL277-$B(OH)_2$-452	ZL277-OH-424
Limitation of detection	32 pg	23 pg	21 pg
Limitation of quantification	160 pg	115 pg	105 pg
Correlation equations	Y=0.00566X+0.000015 R2=0.9927	Y=0.03849X + 0.00003 R2=0.9909	Y=0.53908X+0.000013 R2=9822

Table S6. Detect limitations and correlation equations of belinostat, ZL277-B(OH)$_2$-452, and ZL277-OH-424 for breast tissue during validation.

	Belinostat	ZL277-B(OH)$_2$-452	ZL277-OH-424
Limitation of detection	32 pg	23 pg	21 pg
Limitation of quantification	160 pg	115 pg	105 pg
Correlation equations	Y=0.00561X+0.00010 R2=0.9927	Y=0.03632X + 0.00017 R2=0.9702	Y=0.58106X+0.00015 R2=9935

Table S7. Between-run precision and accuracy of Quality control samples for plasma sample measurement during validation.

	Quality Control(pg)	Mean	SD	% CV	% Acc.
Belinostat	795 pg	940	165	17.5	81.7
	6360 pg	6653	639	9.6	95.4
	25440 pg	24486	1433	5.9	96.2
ZL277-B(OH)$_2$-452	1130 pg	1323	195	14.8	82.9
	9040 pg	8516	783	9.2	94.2
	36160 pg	37690	2940	7.8	95.7
ZL277-OH-424	1060 pg	1226	179	14.6	84.4
	8480 pg	8347	792	9.5	98.4
	33920 pg	35524	2757	7.8	95.3

Table S8. Between-run precision and accuracy of Quality control samples for breast sample measurement during validation.

	Quality Control(pg)	Mean	SD	% CV	% Acc.
Belinostat	795 pg	883	110	12.4	88.9
	6360 pg	6902	491	7.1	91.5
	25440 pg	27030	2116	7.8	93.7
ZL277-B(OH)$_2$-452	1130 pg	1346	115	8.5	80.9
	9040 pg	8730	607	7.0	96.6
	36160 pg	36597	582	1.6	98.8
ZL277-OH-424	1060 pg	1155	148	12.8	91.1
	8480 pg	8875	804	9.1	95.3
	33920 pg	34052	957	2.8	99.6

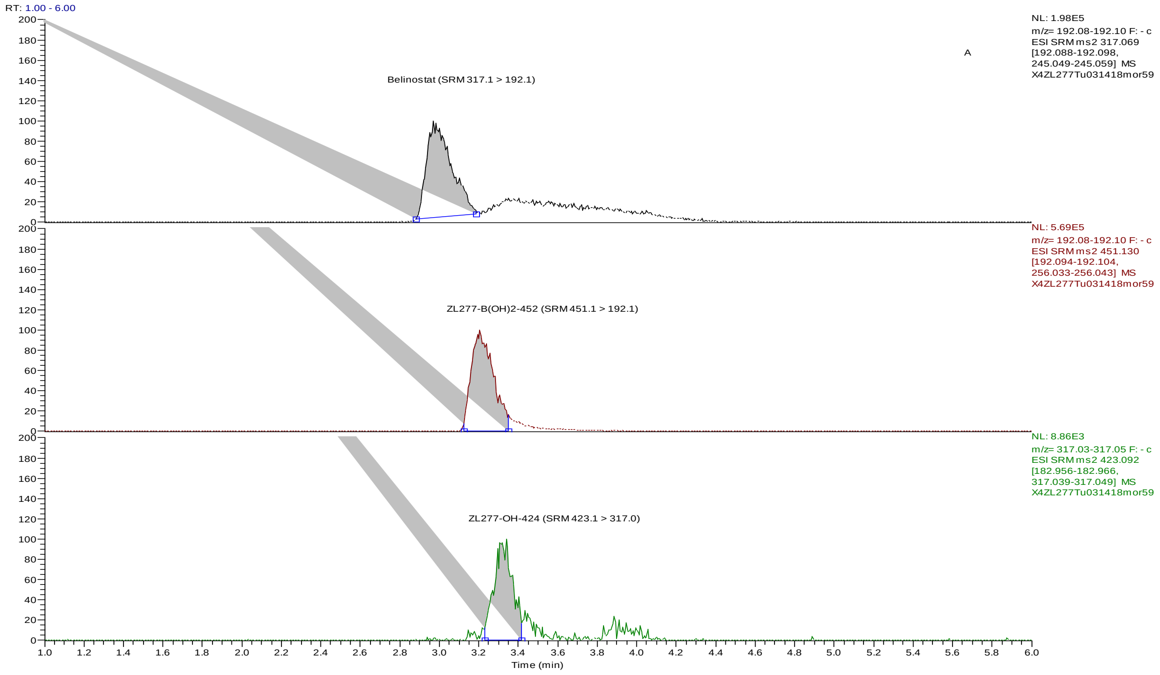
RT: 1.00 - 6.00
A
Belinostat (SRM 317.1 > 192.1)
NL: 1.98E5
m/z= 192.08-192.10 F: - c
ESI SRM ms2 317.069
[192.088-192.098,
245.049-245.059] MS
X4ZL277Tu031418mor59
ZL277-B(OH)2-452 (SRM 451.1 > 192.1)
NL: 5.69E5
m/z= 192.08-192.10 F: - c
ESI SRM ms2 451.130
[192.094-192.104,
256.033-256.043] MS
X4ZL277Tu031418mor59
ZL277-OH-424 (SRM 423.1 > 317.0)
NL: 8.86E3
m/z= 317.03-317.05 F: - c
ESI SRM ms2 423.092
[182.956-182.966,
317.039-317.049] MS
X4ZL277Tu031418mor59
Time (min)

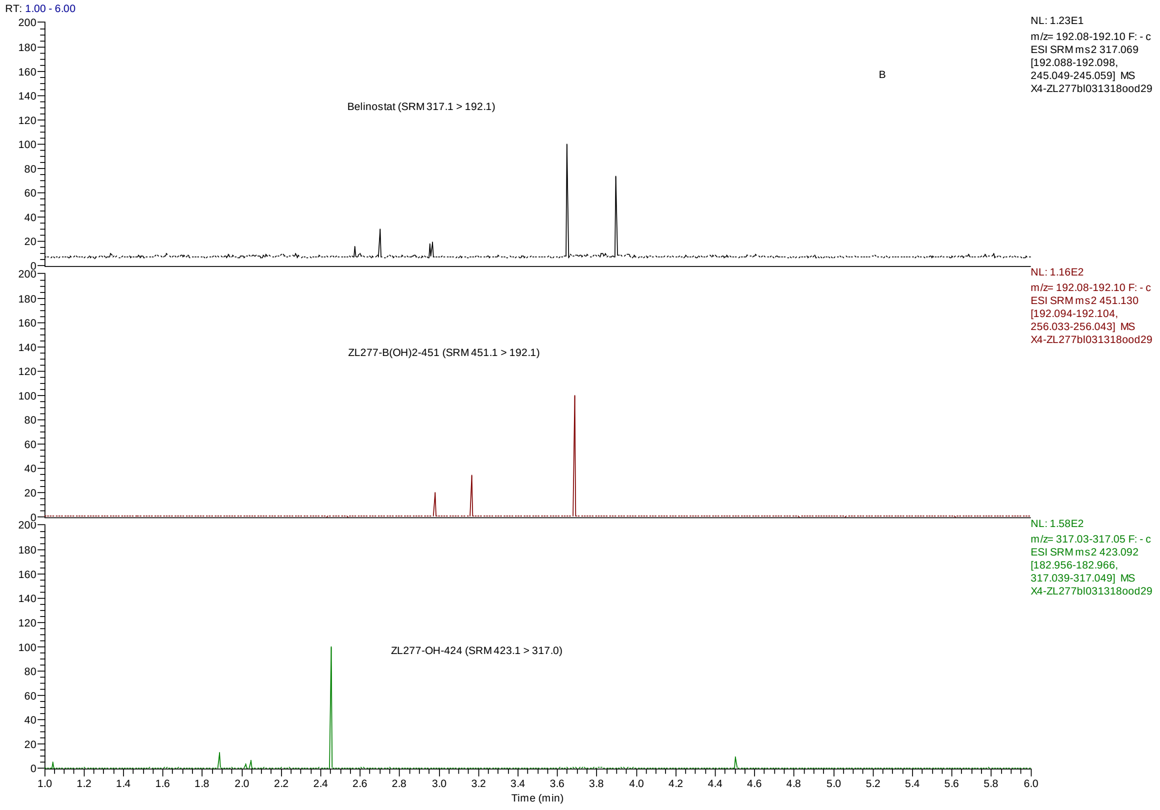
RT: 1.00 - 6.00
B
Belinostat (SRM 317.1 > 192.1)
NL: 1.23E1
m/z= 192.08-192.10 F: - c
ESI SRM ms2 317.069
[192.088-192.098,
245.049-245.059] MS
X4-ZL277bl031318ood29
ZL277-B(OH)2-451 (SRM 451.1 > 192.1)
NL: 1.16E2
m/z= 192.08-192.10 F: - c
ESI SRM ms2 451.130
[192.094-192.104,
256.033-256.043] MS
X4-ZL277bl031318ood29
ZL277-OH-424 (SRM 423.1 > 317.0)
NL: 1.58E2
m/z= 317.03-317.05 F: - c
ESI SRM ms2 423.092
[182.956-182.966,
317.039-317.049] MS
X4-ZL277bl031318ood29
Time (min)

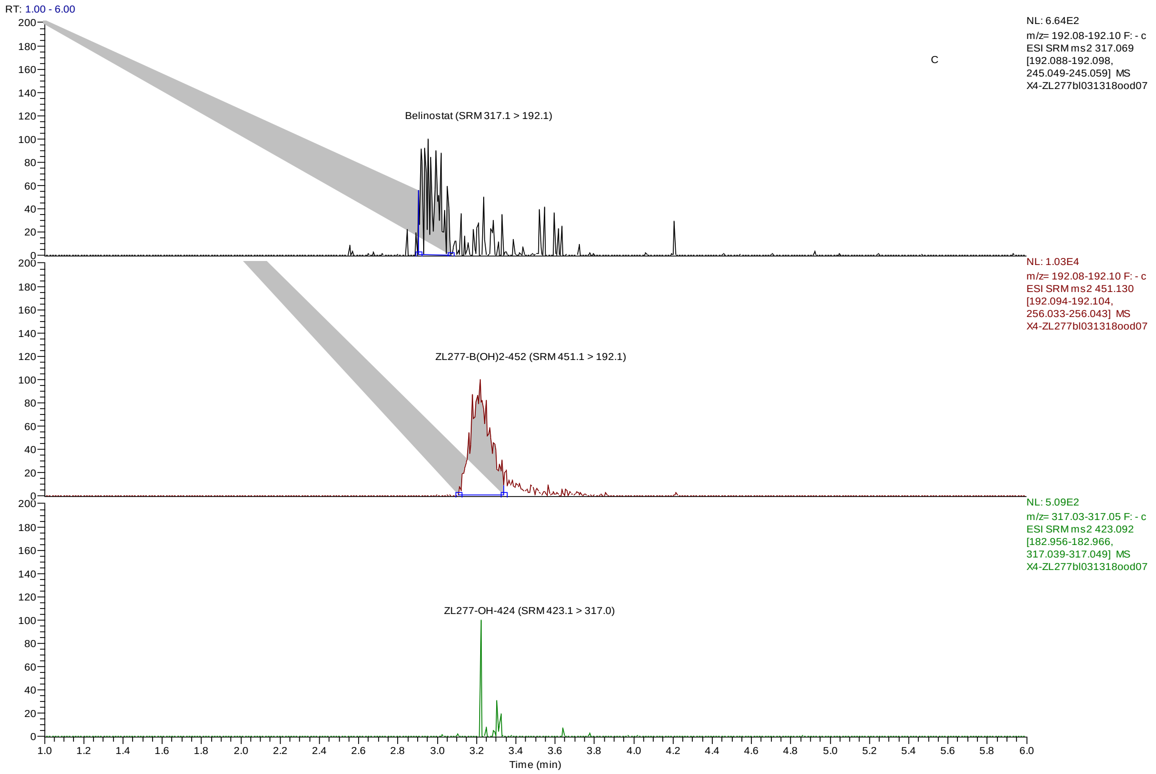

Figure S1. UPLC MS/MS extracted Chromatograms (A) SRM transition of free belinostat, ZL277-$B(OH)_2$-452, and ZL277-OH-424 standard ; (B) a blank mice plasma sample (no belinostat/ZL277); (C) SRM transitions of belinostat, ZL277-$B(OH)_2$-452, and ZL277-OH-424 in mice plasma at 3h after 10mg/kg ZL277 IP injection treatment.

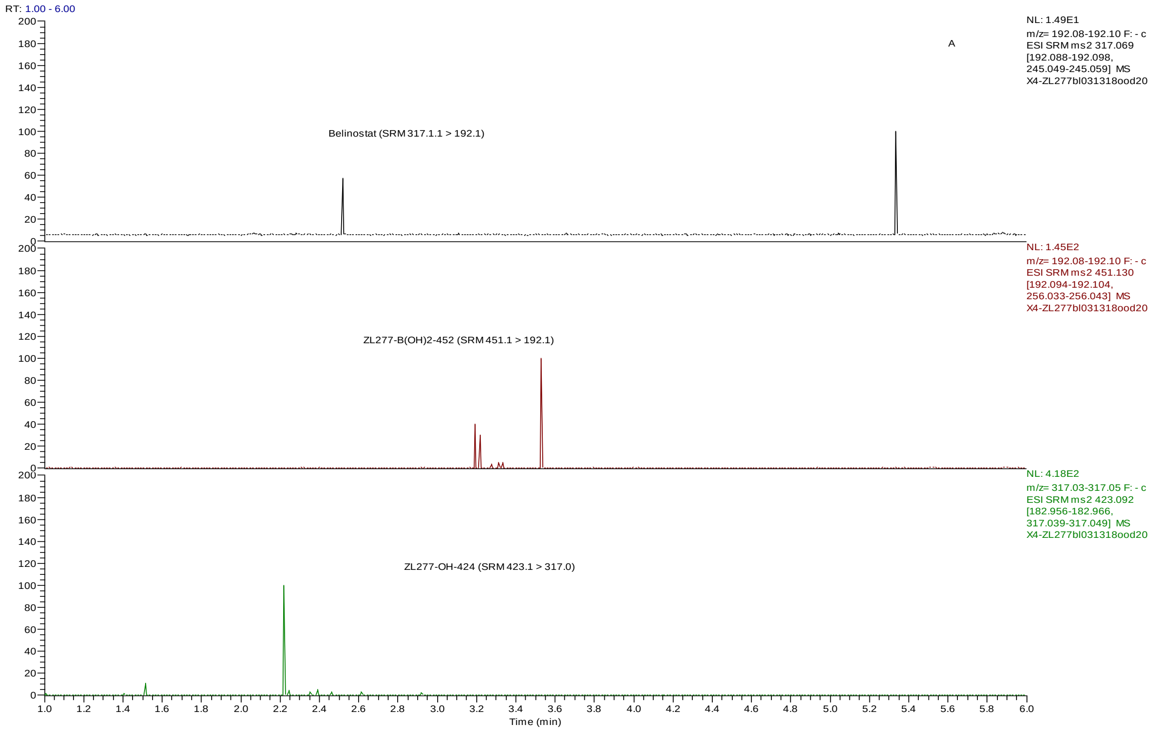

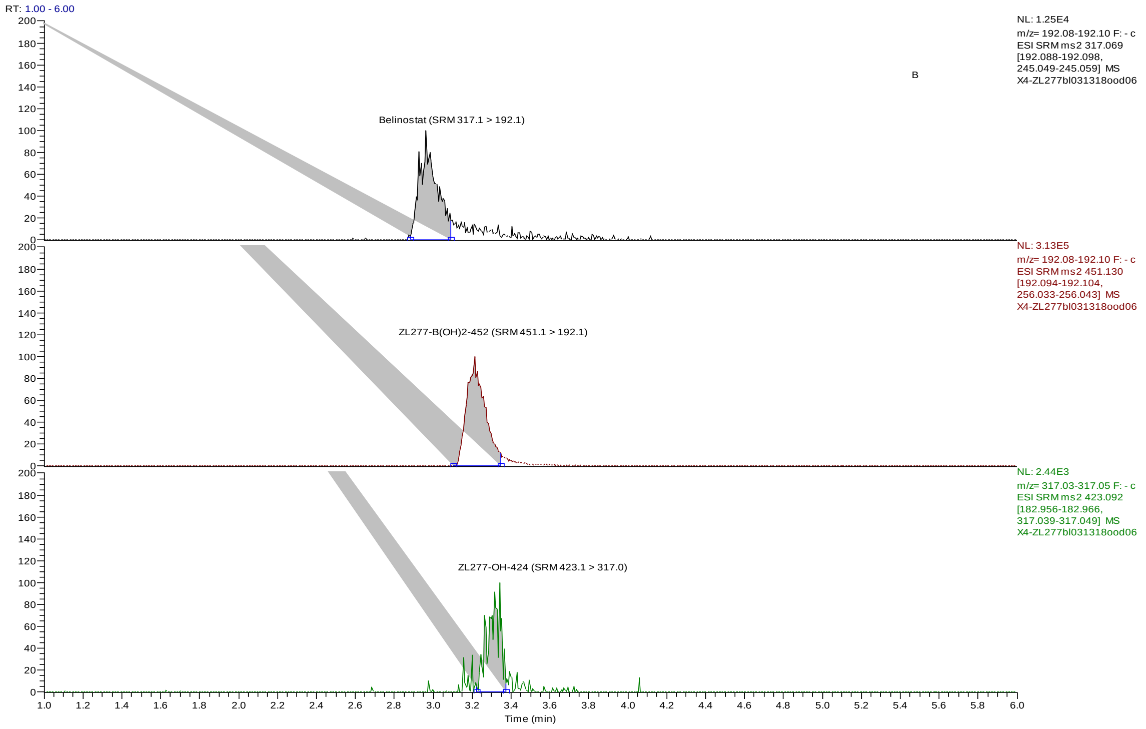

Figure S2. (A) A blank mice breast tumor control sample (without ZL277 or belinostat treatment); (B) SRM transition of belinostat, ZL277-B $(OH)_2$-452, and ZL277-OH-424 in breast tumors at one month after 10 mg/kg ZL277 daily IP injection treatment.

Sample preparation protocols:

(1) Plasma sample preparation: Plasma samples (100 μL) were extracted with methanol. Internal standard (10 μL 0.5 ng/μL trans-tamoxifen-13C2, 15N in methanol), methanol (0.5 mL) were added to each plasma sample. The mixtures were stored at −20 °C overnight. Samples were sonicated for 10 min and then centrifuged on a Heraeus Fresco 21 centrifuge at 14000 rpm. The top liquid layer was dried out and re-suspended in 100 μL of HPLC grade methanol for injection on the TSQ Vantage mass spectrometer instrument.

(2) Feces sample preparation: The collected feces samples were weighed on an OHAUS Explorer balance. The internal standard of trans-tamoxifen-$^{13}C_2$,^{15}N (0.5 ng/μL in methanol) was added to the feces samples based on 10 ng/g feces according to the weight of the feces collected. Methanol was added at the ratio of 4 mL/g feces. Then the samples were homogenized with a Power Gen 125 homogenizer (Fisher Scientific) and stored at -20 °C overnight. After sonicating for 30 min, the samples were centrifuged with a Heraeus Megafuge16R centrifuge at 3000 RPM. The supernatant was dried at room temperature and resuspended with corresponding amount of methanol (200 μL methanol/g feces) for injection on the TSQ Vantage instrument.

(3) Urine sample preparation: Urine samples were thawed at room temperature and vortexed. 200 μL of the sample was transferred to a 1.5 mL Eppendorf microcentrifuge tube followed by adding 200 μL methanol and 10 μL of the 0.5 ng/μL trans-tamoxifen-$^{13}C_2$, ^{15N} in methanol solution. After Vortaxing and centrifuging, the top supernatant was injected on the UPLC-TSQ Vantage mass spectrometer instrument for analysis.
